# Mood and behavioral problems are important predictors of quality of life of nursing home residents with moderate to severe dementia: A cross-sectional study

**DOI:** 10.1371/journal.pone.0223704

**Published:** 2019-12-20

**Authors:** Marinda Henskens, Ilse M. Nauta, Susan Vrijkotte, Katja T. Drost, Maarten V. Milders, Erik J. A. Scherder

**Affiliations:** 1 Department of Clinical Neuropsychology, Vrije Universiteit Amsterdam, Amsterdam, the Netherlands; 2 Department of Neurology, MS Center Amsterdam, Amsterdam UMC, Vrije Universiteit Amsterdam, Amsterdam, the Netherlands; 3 Menzis, Wageningen, the Netherlands; 4 tanteLouise, Bergen op Zoom, the Netherlands; Nathan S Kline Institute, UNITED STATES

## Abstract

**Objectives:**

To examine the predictors associated with quality of life of nursing home residents with dementia, in order to identify which predictors are most important and hold most promise for future intervention studies.

**Methods/Design:**

This cross-sectional analysis of data collected in two intervention trials included 143 participants with moderate to severe dementia who resided in 40 psychogeriatric wards in 13 nursing homes. The outcome measure quality of life was assessed with the Qualidem. Predictors examined were demographic factors, cognition, mood, behavioral problems, and comorbid conditions.

**Results:**

Linear mixed regression analyses showed that all nine domains of quality of life showed independent (negative) associations with either depression, agitation, apathy, or a combination of these predictors. Agitation, apathy, depression, and the presence of neurological disease explained 50% of the variance in total quality of life. Male gender, psychiatric/mood disorders, and having one or more comorbid conditions was associated with worse social relations, while the presence of comorbid neurological diseases was associated with more social isolation and a worse care relationship. The presence of endocrine/metabolic disorders and pulmonary disorders was associated with less restless tense behavior.

**Conclusions:**

Different domains of quality of life showed different associations, confirming the multidimensionality of quality of life in nursing home residents with dementia. Quality of life is independently associated with mood and behavioral problems, comorbid conditions, and gender. This knowledge may help to identify older persons at risk of a lower quality of life, and to offer targeted interventions to improve quality of life.

**Trial registration:**

Dutch Trial registration NTR5641

## Introduction

Nursing home (NH) residents with dementia frequently experience a reduced quality of life (QoL) [1]. QoL is a multidimensional concept for which several definitions have been suggested [2, 3]. QoL in persons with dementia is commonly defined in the context of ‘an integration of cognitive functioning, activities of daily living, social interaction, and psychological well-being’[4]. At present, there is no cure for dementia, and as a result, the focus of dementia care has shifted towards improving or maintaining residents’ optimal QoL. This is confirmed by the growing number of studies that have examined QoL as the primary outcome measure in older persons with dementia [5]. Still, the assessment of QoL in dementia remains complex, as subjective evaluations of QoL may be influenced by cognitive impairment [6]. Moreover, while some researchers recommend observational techniques to measure QoL, others recommend the use of proxy-rated scales, such as the Qualidem for the assessment of QoL in persons with moderate to severe dementia [6, 7]. A multi-method that incorporates self-report, observation, and proxy report has also been recommended [8]. In order to facilitate interventions to improve QoL, it is essential to examine which predictors (e.g. demographic factors, cognition, mood, behavior, and comorbid conditions) are most strongly associated with QoL. Studies that have investigated the predictors associated with QoL of home-dwelling persons with dementia found that depressive symptoms, behavioral disturbances (indicated by the Neuropsychiatric Inventory total score), cognitive impairment, and functional dependence may be associated with a lower QoL [9–12]. However, these results from home-dwelling persons with dementia cannot be generalized to NH residents with more advanced stages of dementia, as these groups differ with regard to their degree of behavioral, functional, and cognitive problems [9].

Several studies have investigated the predictors associated with QoL of NH residents with dementia, yet the results are ambiguous [9, 13–20]. For instance, while depressive symptoms are consistently found to be negatively associated with lower self-rated QoL of NH residents with dementia [9, 15, 17], results are less clear for proxy-rated QoL, for which both the absence and the presence of this association is reported [9, 15–17, 19]. There are indications that the association between depressive symptoms and proxy-rated QoL is stronger in persons with mild to moderate dementia than in those with severe dementia [14, 18]. In contrast to depressive symptoms, the association between behavioral symptoms (e.g. agitation and apathy) and proxy-rated QoL is more evident, suggesting that the presence of behavioral symptoms is related to lower QoL [14–19, 21, 22]. However, although several studies reported that this association was still present when other predictors (e.g. cognition, functional dependence, depression) were taken into account [16–19, 21, 22], other studies found that the association disappeared in these multivariate analyses [14, 15, 23]. The same holds true for the relationship between cognition and QoL: while worse cognitive performance is related to lower QoL [13–21], this association tended to disappear when other predictors, such as behavioral problems (e.g. agitation, apathy, disinhibition), depression, and ADL dependency were taken into account [14, 15, 17, 20, 22].

With regard to demographic predictors (e.g. age and gender) and medical comorbidities, no consistent associations have been found with total QoL [11, 12, 14, 17–19, 21]. To our knowledge, only one study found an association between female gender and a lower total QoL [13] and another study found that higher age, comorbid psychiatric disorders, and pulmonary diseases were negatively associated with the QoL domains social isolation, positive affect, and negative affect, respectively [24]. Overall, these studies do indicate that depressive, behavioral, cognitive, demographic, and comorbid problems are relevant predictors of QoL in NH residents with dementia, but it remains unclear which predictors are most strongly related to QoL.

Moreover, the study by Klapwijk et al. [24] reported that QoL in dementia is associated with different predictors (e.g. comorbid conditions and behavioral and psychological symptoms) across the QoL domains [24]. These results reinforce the broad and multidimensional definition used for QoL, suggesting that QoL should be measured as a multidimensional construct. However, previous research mostly used instruments that assess QoL as a single construct (e.g. Qualid or QoLAD) [13, 15, 17, 19, 21], or considered only a limited number of QoL domains (i.e. ADRQL) [14, 16, 22, 24]. Therefore, the present study builds upon research by focusing on a broader range of predictors as well as examining domains of QoL separately. The aim of this cross-sectional study was to explore in more detail the relative association between demographic factors, cognition, mood, behavioral symptoms, and comorbid conditions with different QoL domains in order to identify the most important predictors in persons with moderate to severe dementia.

## Methods

### Study design

In this cross-sectional study, baseline data were used from two intervention trials which aimed to stimulate movement in NH residents with dementia [25–27]. Details on enrolment as well as the results of the interventions have been described previously [25–27]. In short, participants were recruited from two long-term care organizations in the Netherlands. This study included baseline data from 145 participants with moderate to severe dementia who resided in 40 psychogeriatric wards in 13 nursing home locations. Approval was granted by the scientific and Ethical Review Board (VCWE) of the Faculty of Behavior & Movement Sciences of the Vrije Universiteit Amsterdam (VCWE-2015-183R1), location VU University Amsterdam. Participants’ legal representatives gave written informed consent prior to inclusion.

### Participants

In total, 145 NH residents with dementia were included in the study. First, physiotherapists provided functional mobility assessments for all residents residing at the psychogeriatric wards using the Arjo Mobility Gallery [28]. Only residents with mobility A or B (able to walk and perform ADLs independently with or without assistance) were qualified. Accordingly, geriatricians gave advice about the medical and physical capabilities of the residents. Residents were included if they were aged over 65 years and living on a psychogeriatric ward, with a clinical diagnosis of dementia and a Mini-Mental State Examination (MMSE) [29] score between 1 and 24. Residents were excluded if they were wheelchair bound, had very poor vision, severe cardiovascular problems, unstable diabetes mellitus, or aggressive or runaway behavior. These exclusion criteria were applied due to the intervention studies they were enrolled in [25–27].

### Quality of life

QoL was assessed with the Dutch version of the Qualidem [7, 30]. The questionnaire was completed by the participants’ first responsible caregiver (staff member), if possible together with a qualified nurse in approximately 10 minutes. Moreover, since the nursing staff had no prior experience with the administration of the Qualidem, a trained nursing student administered the Qualidem to the nursing staff by means of an interview. The Qualidem consists of 37 items rated on a four-point rating scale (e.g. never, rarely, sometimes, and frequently). The items describe participants’ behavior in nine domains: (1) care relationship (care relationship with the staff, e.g. conflicts, accepting/appreciating help, criticism on daily routine; range 0–21), (2) positive affect (e.g. being cheerful, satisfied, capable of enjoying things in daily life; range 0–18), (3) negative affect (e.g. being anxious, sad; range 0–9), (4) restless tense behavior (e.g. restless (movements), tense body language; range 0–9), (5) positive self-image (perception of self, e.g. indicating they need help, are not able to do anything or they feel worthless; range 0–9), (6) social relations (contact with other residents, e.g. taking care of others, at ease with company of others, responding positively when approached; range 0–18), (7) social isolation (e.g. rejected by others, rejecting contact with others; range 0–9), (8) feeling at home (feeling at home in the nursing home; range 0–12), and (9) having something to do (e.g. finding things to do without help from others, enjoys helping with chores; range 0–6). For each subscale, a higher score indicated a better QoL. The overall QoL score is the sum of the nine subscales (range 0–111). Higher scores indicate a higher QoL in the different domains. The scales of the Dutch version of the Qualidem are moderately reliable [7, 31] and valid for persons with mild-to-severe dementia [30, 31].

### Predictors of quality of life

#### Global cognitive functioning

Global cognitive functioning was measured with a standardized Dutch translation of the MMSE [29, 32]. Scores of the MMSE range from 0 to 30, where a score below 24 indicates cognitive impairment [33]. The criterion validity of the Dutch version of the MMSE is rated sufficient [34]. There is limited research on the reliability of this version of the MMSE [35].

#### Mood and behavior

Depressive symptoms were measured with the Dutch translation of the Cornell Scale for Depression in Dementia (CSDD) [36, 37]. Scores range from 0 to 38, with higher scores indicating more depressive symptoms [36]. Apathy was measured with the Dutch translation of the Apathy Evaluation Scale-10 (AES-10). Scores range from 10 to 40, with higher scores indicating more apathetic behavior [38]. The frequency of agitated behaviors was measured with the Dutch translation of the Cohen-Mansfield Agitation Inventory (CMAI) [39]. The inventory consists of 29 items representing types of agitated behavior (e.g. hitting, spitting, cursing). Scores range from 29 to 203, with higher scores indicating more agitated behaviors [39, 40]. The depression, apathy, and agitation questionnaires were completed by the participants’ first responsible caregivers together with a qualified nursing staff. The CSDD, AES, and CMAI are valid for elderly with dementia [37, 39, 41].

#### Comorbidities

The comorbidities were derived from the medical records of the participants and were categorized according to the Dutch translation of the Long-Term Care Facility Resident Assessment Instrument (RAI), section I disease diagnosis [42]. The section consists of 44 subcategories belonging to eight categories: (1) endocrine/metabolic diseases, (2) cardiovascular diseases, (3) diseases of the musculoskeletal system, (4) neurological diseases, (5) sensory impairments, (6) psychiatric/mood disorders, (7) pulmonary diseases, and (8) other diseases, including allergies, anemia, cancer, and renal failure. The neurological subcategories ‘Alzheimer’s disease’ and ‘Dementia, other than Alzheimer’s disease’ were not included, as these subcategories are part of the inclusion criteria and therefore were not comorbidities. For each participant, we calculated the number of categories affected, as well as the total number of comorbidities. In addition, comorbidity was categorized into four levels: no comorbidity, mild (1–2 conditions), moderate (3–4 conditions), and severe (>5 conditions) comorbidity.

### Statistical analysis

All statistical analyses were performed using IBM Statistical Package for the Social Sciences (SPSS) 25.0 [43]. The assumptions of linear mixed regression were confirmed for all models.

Each comorbidity was an individual variable, which was dichotomized (0 = absent, 1 = present). Participants with missing data on one or more variables were excluded from the analyses. Due to the clustered nature of our sample, a two-level regression model was used (e.g. participants nested in NHs). The maximum likelihood method was used to examine the possible superiority of random intercept and slope models. The model of the best fit was used for the final analyses. Simple and multiple linear mixed regression models were performed to identify associations between predictors and QoL. First, simple linear mixed regression analyses were performed to screen for candidate predictors of quality of life. Second, to ensure that all predictive variables were included in the multiple linear mixed models, variables that had a relation with the Qualidem with *p*-values lower than 0.10 were included as potentially predictive variables in the multiple linear mixed regression analyses. Backwards regressions were run to remove non-significant variables (*p*-values higher than 0.05) from the final models.

## Results

### Study population

An overview of the group characteristics is given in Table 1. Of the 145 participants included in the study, one participant had missing data on the Qualidem and one participant had missing data regarding comorbid conditions, leaving 143 participants for the present analysis (77.60% female). The age ranged from 69 to 100 years (mean age *=* 85.65). The average MMSE score indicates a moderately severe stage of cognitive impairment (MMSE = 11.10, SD = 5.45) [44]. On average, participants had a total of 3.36 comorbid disorders. The 5 most frequent comorbid conditions across the sample were: hypertension (45.50%), cataract (30.10%), renal failure (26.60%), diabetes mellitus (21.70%), and arrhythmias (17.50%). In total, 5.60% of the sample did not have any comorbid condition, 25.90% had mild comorbidity (1–2 conditions), 42.70% had moderate comorbidity (3–4 conditions), and 25.90% had severe comorbidity (≥5 conditions).

**Table 1:** Descriptive statistics for demographic and clinical predictors (N = 143).

Variables	Participants
**Age, mean *(SD)***	85.65 (6.09)
**Gender (female), *n* (%)**	111 (77.60)
**Diagnosis, *n* (%)**	
Alzheimer’s disease (AD)	62 (43.40)
Vascular dementia (VD)	19 (13.30)
Mixed AD and VD	12 (8.40)
Dementia not otherwise specified	47 (32.90)
Korsakoff	2 (1.40)
Unknown	1 (0.60)
**Cognitive function (MMSE), mean (*SD)***	11.10 (5.45)
**Agitation (CMAI), mean (*SD)***	48.78 (14.57)
**Apathy (AES-10), mean (*SD)***	26.83 (7.94)
**Depression (CSDD), mean (*SD)***	8.07 (4.62)
**Qualidem, mean (*SD)***	
Care relationship	14.74 (4.10)
Positive affect	14.68 (3.30)
Negative affect	5.69 (2.30)
Restless tense behavior	4.59 (2.48)
Positive self-image	6.43 (2.18)
Social relations	12.24 (3.94)
Social isolation	6.19 (2.04)
Feeling at home	8.92 (2.60)
Having something to do	2.85 (1.92)
**Total QoL, mean (*SD)***	76.31 (15.22)
**Total number of comorbid categories affected, mean (*SD)***	2.62 (1.32)
**Total number of comorbidities, mean (*SD)***	3.36 (1.86)
**Comorbidities, *n*(%)**	
Endocrine/metabolic disease^a^	44 (30.80)
Heart/cardiovascular disease^b^	105 (73.40)
Diseases of musculoskeletal system^c^	50 (35.00)
Neurological diseases ^d^	35 (24.50)
Sensory impairment^e^	52 (36.40)
Psychiatric/mood disorders^f^	19 (13.30)
Pulmonary^g^	13 (9.10)
Other^h^	57 (39.90)

^a^ Diabetes mellitus, hypothyroidism, hyperthyroidism;

^b^ Arteriosclerotic disease, arrhythmias, heart failure, deep vein thrombosis, hypertension, hypotension, peripheral vascular disease, other;

^c^ Rheumatic diseases, hip fracture, amputation, osteoporosis, pathologic bone fracture;

^d^ Aphasia, cerebral palsy, stroke, hemiplegia/hemiparesis, paraplegia, multiple sclerosis, Parkinson disease, seizures, transient cerebral ischemia, traumatic brain injury, quadriplegia;

^e^ Cataract, diabetic retinopathy, glaucoma, macular degeneration;

^f^ Anxiety disorder, depression, manic depression, schizophrenia;

^g^ Asthma, emphysema/COPD;

^h^ Allergies, anemia, cancer, renal failure

**Abbreviations.** MMSE, mini-mental state examination; QoL, quality of life; CMAI, Cohen-Mansfield Agitation Inventory; CSDD, Cornell Scale for Depression in Dementia; AES-10, apathy evaluation scale-10.

### Simple linear mixed regression analyses

#### Demographics

Results of the simple linear mixed regression analyses are shown in Tables 2 and 3. Male gender was associated with having worse social relations (*p* = 0.03), more social isolation (*p* = 0.06), and less negative affect (*p* = 0.08). Higher age was associated with more positive affect (*p* = 0.02).

**Table 2:** Results of the simple linear mixed regression analyses with Qualidem as dependent variable.

	Care relationship		Positive affect		Negative affect		Restless tense behavior		Positive self-image	
	B (95%CI)	β	B (95%CI)	β	B (95%CI)	β	B (95%CI)	β	B (95%CI)	β
**Age**	0.06 (-0.05, 0.17)	0.09	0.11 (0.02, 0.20)*	0.20	0.03 (-0.04, 0.09)	0.08	0.05 (-0.02, 0.12)	0.12	-0.02 (-0.08, 0.04)	-0.06
**Gender, female**	0.52 (-1.10, 2.13)	0.05	0.71 (-0.59, 2.01)	0.09	-0.81 (-1.71, 0.10)	-0.15	0.08 (-0.91, 1.07)	0.01	-0.69 (-1.55, 0.17)	-0.13
**Cognitive function (MMSE)**	-0.06 (-0.18, 0.07)	-0.08	0.14 (0.05, 0.24)**	0.23	0.06 (-0.01, 0.13)	0.14	0.08 (0.00, 0.15)*	0.18	0.05 (-0.01, 0.12)	0.13
**CSDD**	-0.10 (-0.14, -0.06)**	-0.36	-0.08 (-0.12, -0.05)**	-0.35	-0.05 (-0.07, -0.02)**	-0.32	-0.11 (-0.13, -0.08)**	-0.65	-0.04 (-0.07, -0.02)**	-0.27
**AES-10**	0.02 (-0.07, 0.10)	0.04	-0.22 (-0.28, -0.17)**	-0.53	-0.01 (-0.06, 0.04)	-0.03	-0.03 (-0.08, 0.03)	-0.10	-0.06 (-0.10, -0.01)*	-0.22
**CSDD**	-0.29 (-0.43, -0.15)**	-0.33	-0.34 (-0.44, -0.23)**	-0.48	-0.28 (-0.35, -0.21)**	-0.56	-0.23 (-0.31, -0.15)**	-0.43	-0.17 (-0.24, -0.10)**	-0.36
**Endocrine/metabolic**	0.78 (-0.67, 2.24)	0.09	0.21 (-0.97, 1.39)	0.03	0.36 (-0.47, 1.18)	0.07	0.88 (0.00, 1.76)*	0.16	0.30 (-0.48, 1.08)	0.06
Cardiovascular diseases	-0.64 (-2.16, 0.89)	-0.07	-0.16 (-1.40, 1.07)	-0.02	0.22 (-0.65, 1.08)	0.04	0.16 (-0.77, 1.10)	0.03	0.21 (-0.61, 1.03)	0.04
Musculoskeletal disorder	-0.45 (-1.86, 0.97)	-0.05	0.27 (-0.88, 1.41)	0.04	-0.53 (-1.33, 0.27)	-0.11	0.13 (-0.73, 0.99)	-0.03	-0.48 (-1.24, 0.27)	-0.11
**Neurological diseases**	-2.17 (-3.69, -0.64)*	-0.23	-1.52 (-2.8, -0.28)	-0.20	0.00 (-0.89, 0.89)	0.00	-0.75 (-1.70, 0.20)	-0.12	0.06 (-0.78, 0.90)	0.01
Sensory impairment	0.06 (-1.36, 1.47)	0.01	0.16 (-0.98, 1.31)	0.02	-0.14 (-0.93, 0.65)	-0.03	-0.60 (-1.45, 0.25)	-0.12	-0.41 (-1.16, 0.34)	-0.09
**Psychiatric/mood disorder**	-0.30 (-2.28, 1.68)	-0.03	-0.99 (-2.59, 0.61)	-0.10	-0.43 (-1.55, 0.70)	-0.06	-0.63 (-1.83, 0.58)	-0.09	-1.21 (-2.26, -0.17)*	-0.19
**Pulmonary**	0.56 (-1.79, 2.91)	0.04	0.30 (-1.60, 2.19)	0.03	0.26 (-1.07, 1.59)	0.03	1.38 (-0.04, 2.79)	0.16	0.28 (-0.98, 1.54)	0.04
**Other**	-0.80 (-2.18, 0.57)	-0.10	-0.31 (-1.43, 0.81)	-0.05	0.76 (-0.01, 1.53)	0.16	0.56 (-0.28, 1.39)	0.11	0.22 (-0.52, 0.95)	0.05
Total comorbidities	-0.22 (-0.59, 0.14)	-0.10	-0.11 (-0.40, 0.19)	-0.06	0.11 (-0.10, 0.31)	0.09	-0.02 (-0.24, 0.20)	-0.02	-0.07 (-0.27, 0.12)	-0.06
**No comorbidities vs >1**	3.75 (0.88, 6.61)*	0.21	1.85 (-.51, 4.20)	0.13	-.06 (-1.73, 1.60)	-0.01	0.30 (-1.49, 2.09)	0.03	0.12 (-1.45, 1.70)	0.01

Qualidem: higher (sub)scores indicate higher QoL; Bold values represent predictor variables to be entered in the final multiple linear regression analyses (*p-*values < 0.10).

**Abbreviations**: MMSE, mini-mental state examination; CMAI, Cohen-Mansfield Agitation Inventory; CSDD, Cornell Scale for Depression in Dementia; AES-10, apathy evaluation scale-10; B, unstandardized regression coefficient; β, standardized regression coefficient.

**p < .005

* p < .05,

**Table 3:** Results of the simple linear mixed regression analyses with Qualidem as dependent variable.

	Social relations		Social isolation		Feeling at home		Having something to do		Total QoL	
	B (95%CI)	β	B (95%CI)	β	B (95%CI)	β	B (95%CI)	β	B (95%CI)	β
**Age**	0.04 (-0.06, 0.15)	0.06	0.01 (-0.05, 0.06)	0.03	0.04 (-0.03, 0.11)	0.09	-0.02 (-0.07, 0.03)	-0.06	0.29 (-0.11, 0.69)	0.12
**Gender, female**	1.65 (0.18, 3.12)*	0.18	0.76 (-0.02, 1.54)	0.16	-0.42 (-1.45, 0.61)	-0.07	-0.03 (-0.74, 0.68)	0.01	1.86 (-3.99, 7.71)	0.05
**Cognitive function (MMSE)**	0.22 (0.10, 0.33) **	0.30	0.05 (-0.01, 0.12)	0.13	-0.04 (-0.12, 0.04)	-0.08	0.11 (0.05, 0.16)**	0.31	0.61 (0.15, 1.07)*	0.22
**Agitation (CMAI)**	-0.02 (-0.07, 0.02)	-0.07	-0.05 (-0.07, -0.03)**	-0.36	-0.06 (-0.09, -0.03)**	-0.34	0.01 (-0.02, 0.03)	0.08	-0.50 (-0.65, -0.36)**	-0.48
**Apathy (AES-10)**	-0.27 (-0.34, -0.21)**	-0.54	-0.03 (-0.07, 0.01)	-0.12	0.01 (-0.05, 0.06)	0.03	-0.15 (-0.18, -0.12)**	-0.62	-0.75 (-1.04, -0.46)**	-0.39
**Depression (CSDD)**	-0.18 (-0.32, -0.05)*	-0.21	-0.14 (-0.21, -0.07)**	-0.32	-0.13 (-0.23, -0.04)*	-0.23	-0.05 (-0.11, 0.02)	-0.12	-1.78 (-2.23, -1.33)**	-0.54
**Endocrine/metabolic**	0.13 (-1.22, 1.47)	0.02	0.31 (-0.40, 1.02)	0.07	0.43 (-0.50, 1.37)	0.08	0.11 (-0.54, 0.75)	0.03	3.13 (-2.13, 8.40)	0.10
Cardiovascular diseases	0.09 (-1.33, 1.50)	0.01	0.50 (-0.24, 1.25)	0.11	0.39 (-0.59, 1.36)	0.07	-0.22 (-0.90, 0.45)	0.05	0.50 (-5.05, 6.05)	0.02
Musculoskeletal disorder	0.51 (-0.80, 1.82)	0.06	0.35 (-0.34, 1.04)	0.08	-0.51 (-1.41, 0.39)	-0.09	-0.28 (-0.90, 0.35)	-0.07	-1.22 (-6.36, 3.91)	-0.04
**Neurological diseases**	-1.65 (-3.08, 0.22)*	-0.18	-1.07 (-1.83, -0.31)*	-0.23	-0.45 (-1.46, 0.56)	-0.07	-0.34 (-1.04, 0.36)	-0.08	-7.71 (-13.34, -2.08)*	-0.22
Sensory impairment	0.30 (-1.03, 1.63)	0.04	0.40 (-0.30, 1.10)	0.10	-0.17 (-1.07, 0.73)	-0.03	0.13 (-0.51, 0.76)	0.03	-0.61 (-5.81, 4.59)	-0.02
**Psychiatric/mood disorder**	-2.86 (-4.62, -1.10)**	-0.25	-0.68 (-1.64, 0.28)	-0.11	0.81 (-0.46, 2.07)	0.11	-0.21 (-1.07, 0.66)	-0.04	-6.44 (-13.52, 0.65)	-0.15
**Pulmonary**	-1.61 (-3.76, 0.55)	-0.12	0.32 (-0.82, 1.47)	0.05	-0.17 (-1.68, 1.33)	-0.02	0.17 (-0.87, 1.20)	0.03	1.31 (-7.20, 9.83)	0.02
**Other**	0.37 (-1.65, 0.92)	0.05	0.29 (-0.39, 0.97)	0.07	0.35 (-0.53, 1.24)	0.07	-0.31 (-0.93, 0.31)	-0.08	0.49 (-4.58, 5.56)	0.02
Total comorbidities	-0.21 (-0.55, 0.12)	-0.10	0.05 (-0.13, 0.23)	0.05	0.04 (-0.20, 0.27)	0.03	-0.11 (-0.27, 0.05)	-0.11	-0.57 (-1.89, 0.74)	-0.07
**No comorbidities vs >1**	3.43 (0.79, 6.08)*	0.20	1.11 (-0.32, 2.54)	0.13	-1.39 (-3.25, 0.47)	-0.12	0.78 (-0.50, 2.06)	0.09	10.32 (-0.18, 20.8)	0.16

Qualidem: higher (sub)scores indicate higher QoL; Bold values represent predictor variables to be entered in the final multiple linear regression analyses (*p-*values < 0.10).

**Abbreviations**: MMSE, mini-mental state examination; CMAI, Cohen-Mansfield Agitation Inventory; CSDD, Cornell Scale for Depression in Dementia; AES-10, apathy evaluation scale-10; B, unstandardized regression coefficient; β, standardized regression coefficient.

**p < .005

* p < .05,

#### Mood and behavioral symptoms

A higher level of agitation was associated with a lower QoL with regard to the domains care relationship, positive affect, negative affect, restless tense behavior, positive self-image, social isolation, feeling at home, and total QoL (all *p*’s < 0.005; Tables 2 and 3). A higher level of apathy was associated with a lower QoL with regard to the domains positive self-image (*p =* 0.01), positive affect, social relations, having something to do, and total QoL (all *p*’s < 0.005). A higher level of depression was associated with a lower QoL with regard to all domains (all *p’s* < 0.05), except for the domain ‘having something to do’ (*p* = 0.14).

#### Cognitive function

Higher cognitive function was associated with a higher QoL in the domains positive affect, social relations, having something to do (all *p’s* < 0.005), restless tense behavior, total QoL (*p*’s < 0.05), negative affect, and social isolation (*p*’s < 0.10).

#### Comorbidities

Having no medical comorbidities was associated with having a better care relationship, social relations (*p’s* < 0.05), and total QoL (*p* = 0.05). The presence of neurological diseases was associated with a lower positive affect (*p* = 0.02), worse care relationship and social relations, more social isolation, and a lower total QoL (all *p*’s < 0.05). The presence of psychiatric/mood disorders was associated with a lower QoL with regard to positive self-image (*p* = 0.02), social relations (*p* = 0.002), and total QoL (*p* = 0.07). The presence of pulmonary diseases was associated with less restless tense behavior (*p* = 0.06).

### Multiple linear mixed regression analyses

Results of the multiple linear mixed regression analyses showed that all nine domains of QoL showed negative associations with either depression (range β = -0.56 to β = -0.18), agitation (range β = -0.53 to β = -0.18), apathy (range β = -0.62 to β = -0.26) or a combination of these predictors (Table 4), suggesting that the presence of mood and behavioral disorders is associated with a lower QoL in all domains. Moreover, depression (β = -0.33), apathy (β = -0.26), and agitation (β = -0.30), together with comorbid neurological diseases (β = -0.15) explained 50% of the variance in total QoL, with depression being the most important predictor. Apathy (β = -0.52), male gender (β = 0.20), comorbid psychiatric/mood disorders (β = -0.21), and having no comorbid conditions (β = 0.16) together explained 47% of the variance in social relations. Comorbid neurological diseases (other than dementia) were negatively associated with total QoL, and the QoL domains care relationship (β = -0.20) and social isolation (β = -0.22). Comorbid endocrine/metabolic disorders (β = 0.14) and pulmonary disorders (β = 0.14) showed positive associations with restless tense behavior, suggesting that the presence of comorbid endocrine/metabolic disorders and pulmonary disorders is associated with less restless tense behavior.

**Table 4:** Multiple linear mixed regression analysis of associations between Qualidem scales and predictors (n = 143).

		B (95%CI)	β	*p*	*Adjusted R^2^*
**Care relationship**					0.21
Agitation (CMAI)	-0.07 (-0.12, -0.03)	-0.25	0.003	
Neurological diseases	-1.87 (-3.29, -0.44)	-0.20	0.01	
Depression (CSDD)	-0.16 (-0.31, -0.01)	-0.18	0.04	
**Positive affect**					0.43
Apathy (AES-10)	-0.19 (-0.24, -0.13)	-0.46	<0.001	
Depression (CSDD)	-0.20 (-0.31, -0.10)	-0.28	<0.001	
Agitation (CMAI)	-0.04 (-0.07, -0.01)	-0.18	0.02	
**Negative affect**					0.36
Depression (CSDD)	-0.27 (-0.34, -0.20)	-0.56	<0.001	
**Restless tense behavior**					0.43
Agitation (CMAI)	-0.09 (-0.11, -0.06)	-0.53	<0.001	
Depression (CSDD)	-0.10 (-0.18, -0.02)	-0.19	0.01	
Endocrine/metabolic	0.74 (0.07, 1.42)	0.14	0.03	
Pulmonary	1.19 (0.11, 2.28)	0.14	0.03	
**Positive self-image**					0.14
Depression (CSDD)	-0.17 (-0.24, -0.10)	-0.36	<0.001	
**Social relations**					0.47
Apathy (AES-10)	-0.26 (-0.32, -0.19)	-0.52	<0.001	
Psychiatric disorder	-2.17 (-3.61, -0.73)	-0.19	0.003	
Male gender	1.87 (0.71, 3.03)	0.20	0.002	
No comorbidities vs >1	2.69 (0.57, 4.80)	0.16	0.01	
**Social Isolation**					0.25
Agitation (CMAI)	-0.05 (-0.07, -0.03)	-0.36	0.001	
Neurological diseases	-1.05 (-1.74, -0.35)	-0.22	0.004	
**Feeling at home**					0.10
Agitation (CMAI)	-0.06 (-0.09, -0.03)	-0.34	<0.001	
**Having something to do**					0.52
Apathy (AES-10)	-0.15 (-0.18, -0.12)	-0.62	<0.001	
**Total QoL**					0.50
Depression (CSDD)	-1.11 (-1.57, -0.66)	-0.33	<0.001	
Agitation (CMAI)	-0.31 (-0.45, -0.17)	-0.30	<0.001	
Apathy (AES-10)	-0.49 (-0.73, -0.25)	-0.26	<0.001	
Neurological diseases	-5.53 (-9.76, -1.30)	-0.15	0.01	

Qualidem: higher (sub)scores indicate higher QoL; Outcome variables: care relationship, positive affect, negative affect, restless tense behavior, positive self-image, social relations, social isolation, feeling at home, having something to do, total QoL; Predictor variables: agitation, apathy, depression, neurological disease, endocrine/metabolic disease, pulmonary disease, psychiatric disorder, no comorbidities vs >1 comorbidity, male gender.

**Abbreviations.** B, unstandardized regression coefficient; β, standardized regression coefficient; CMAI, Cohen-Mansfield Agitation Inventory; CSDD, Cornell Scale for Depression in Dementia; AES-10, apathy evaluation scale-10; QoL, quality of life.

## Discussion

This study investigated a broad range of predictors (e.g. demographic factors, cognition, mood, behavior, and comorbidities) of QoL in NH residents with moderate to severe dementia, in order to identify which predictors are most important and hold most promise for future intervention studies. Our results confirm the multidimensionality of QoL, as different domains of QoL were associated with different predictors. Previous studies that investigated the individual contribution of multiple predictors on QoL were generally limited to examining QoL as a single construct or considered only a limited number of domains of QoL [13–17, 19, 21, 22, 24].

Our study showed that agitation, apathy, and depression were the predictors most consistently and strongly associated with QoL. Specifically, agitation was associated with a worse care relationship, lower positive affect, more restless tense behavior, a larger sense of social isolation, and less sense of feeling at home. Apathy was associated with a lower positive affect, worse social relations, and less sense of having something to do. Apathy manifests as a lack of motivation and interest, taking initiative, and goal-directed behavior. This may negatively influence a person’s ability to take initiative in participating in social contact with others, to perform self-care activities, as well as to find things to do without help from others [45]. Depressive symptoms were associated with a worse care relationship, lower positive affect, lower positive self-image, more negative affect, and more restless tense behavior. One explanation for the association between depressive symptoms and several domains of QoL is that depressive symptoms can distort the perception of realty, due to which persons may see themselves in a negative way and interpret events unfavorably [46]. Similarly, the presence of comorbid psychiatric disorders (i.e. diagnosed depression) was associated with worse social relations. These robust associations reported in this study between mood and behavior and all domains of QoL support and extend previous literature. Klapwijk et al. [24] investigated six QoL domains, and also found that the presence of behavioral and psychological symptoms as one overall predictor negatively related with care relationship, positive affect, negative affect, restless tense behavior, social relations, and social isolation. Importantly, our study highlighted that apathy, depression, and agitation were all unique correlates of QoL and explained a large proportion of the variance in total QoL. This indicates the relevance of treating each of these mood and behavioral problems, although no conclusions with regard to causality can be drawn from our study. In addition, our results indicate that mood and behavior are more important correlates of QoL than cognition, demographic factors, and comorbid conditions. Taken together, these findings suggest that interventions aimed at improving mood and behavioral problems may also benefit all domains of QoL of NH residents with moderate to severe dementia. Especially non-pharmacological interventions (e.g. sensory interventions) and care approaches (e.g. training staff in communication, person-centered care skills, addressing unmet needs) are promising interventions to reduce mood and behavioral problems in dementia [47, 48].

In addition to mood and behavioral problems, our study showed that some comorbidities (i.e. neurological, pulmonary, and endocrine/metabolic diseases) were also important predictors of QoL. Importantly, persons without comorbidities were more likely than persons with one or more comorbidities to have better social relations. One explanation for this finding could be that the more comorbidities a person has, the greater their impairments in cognitive functioning [49]. Impaired cognitive functioning in turn negatively influences social participation [50]. When looking specifically at comorbidity categories, our study showed that the presence of neurological diseases (i.e. stroke and TIA) was associated with a worse care relationship, more social isolation, and a lower total QoL. This is also found in the general population [51], where it has been suggested that the degree of physical, cognitive, and emotional impairment following, for instance, stroke, limits the amount of social interaction a person is able to undertake, thereby increasing social isolation [52]. The presence of endocrine/metabolic disorders (i.e. hypo/hyperthyroidism and diabetes) and pulmonary disorders (i.e. COPD and asthma) was associated with less restless tense behavior (e.g. repeatedly standing up and walking around, repeatedly moving legs). This is an interesting finding, and to our knowledge, no previous studies have reported this association. The presence of restless tense behavior might reflect exercise capacity and physical ability. It has been established previously that persons with pulmonary diseases and endocrine/metabolic disorders have limited exercise capacity and are less physically active [53, 54]. Our findings might suggest that certain comorbid diseases limit physical possibilities which may result in less restless behavior.

Although the total number of comorbid disorders seemed to have little impact on QoL in our study, which is consistent with results from a previous study [24], the observed associations between specific comorbid conditions and domains of QoL might be clinically relevant, as these associations could point at a possible benefit of symptom control and timely treatment of certain comorbid conditions (e.g. neurological diseases) for QoL. This association between comorbid disorders and QoL has also found in the general population [55]. Comorbid diseases are frequently under-diagnosed and incorrectly treated in persons with dementia [56], and a higher number of comorbid conditions may lead to polypharmacy [57] and increased pain [58]. The experienced pain results in more agitation which in turn can negatively affect QoL [58]. On the other hand, effective management of comorbid conditions has been found to reduce agitation, making it a crucial aspect of QoL management, especially with regard to care relationship, social isolation, and total QoL [59]. Indeed, a recent study found that an intervention which benefits challenging behavior, pain, and the use of medication, can also lead to improvements in domains of QoL (e.g. restless tense behavior and social isolation) in NH residents with advanced dementia [60].

Our study showed that male gender was an independent predictor of having worse social relations. Even though we did not find gender differences in other domains, it may indicate that men have a larger chance of having a lower QoL. However, to our knowledge, no robust associations have previously been found between gender and QoL [11, 12, 14, 17–19, 21]. In contrast to our finding, a few studies reported female gender to be associated with a lower total QoL [13, 61, 62]. It is conceivable that being female may be associated with a lower total QoL, considering that depression, which is more common in women [63], negatively influences several aspects of QoL (e.g. care relationship, positive affect, negative affect, restless tense behavior, positive self-image). However, the fact that male gender arose as an independent predictor of worse social relations in the current study is feasible considering that women generally perform better on aspects concerning social relationships [64, 65]. Men traditionally show more behaviors (e.g. aggressiveness or competitiveness) which are considered to be barriers to forming relationships [64]. Although no firm conclusions with regard to gender can be drawn, the findings may imply that male gender, psychiatric disorders, apathy, and having one or more comorbid conditions increases a person’s vulnerability for having worse social relations. Knowledge regarding the predictors associated with social relations is important, as social relations have been associated with various health outcomes, including mortality risk, functional and cognitive functions, and well-being [66, 67]. Considering that apathy, which is manifested by a loss of self-initiated behavior such as starting a conversation [68], emerged as the most important predictor of social relations, staff and caregivers may play a vital role in initiating and facilitating social relations in the NH setting.

Global cognition (as measured by the MMSE) showed positive associations with total QoL and six domains of QoL. However, these associations were not maintained when other predictors (especially mood and behavioral problems) were also taken into account. Previous studies report contradictory findings regarding multivariate associations between MMSE and QoL [13–19, 21, 22]. These inconsistencies may be explained by the large variation in assessment instruments used to measure QoL and cognition (Qualidem vs. QoLAD, Qualid, or ADRQL; MMSE vs. SIB), as well as the diverse predictors accounted for in the prediction models. Importantly, previous studies that did find a unique effect of cognition on QoL did not account for apathy, agitation, and depression as separate predictors [13, 18, 21]. Our study highlights that these neuropsychiatric problems should be taken into account as individual predictors, and that these seem to have a larger contribution to QoL than the level of cognitive impairment.

A few limitations of the study must be considered. First, we did not consider *every* predictor that may have been associated with QoL. For instance, it is possible that pharmacological treatment or family support affect QoL [14]. Still, we did investigate a broad range of predictors that seemed to have an important influence. Second, the cross-sectional design of the study does not allow drawing conclusions on causality. Future longitudinal studies may be able to investigate which factors underlie future decreases in QoL. Third, we used only a proxy-rated assessment of QoL, which may be influenced by the nature of the staff’s relationship with the resident [21]. Even though proxy-ratings are the recommended method for assessment of QoL in persons with moderate to severe dementia [6, 7], such ratings should be interpreted with caution, as self and proxy-rated QoL ratings show different associations with patient characteristics, and staff tend to underestimate the QoL of persons with dementia [21]. Therefore, in future research it may be valuable to use both self and proxy methods of assessment when identifying predictors associated with QoL of NH residents with moderate to severe dementia [69]. Still, self-assessed methods are faced with difficulties in this patient group, and therefore, we may have to accept this limitation. Fourth, the generalizability of the study was limited by several factors, including the sample size, the limited number of NH’s and psychogeriatric wards, the exclusion criteria, and the relatively high percentage of participants diagnosed with ‘dementia not otherwise specified’. The use of several outcomes with several predictors may have led to some results being the result of chance. Last, no distinction was made between subtypes of dementia, while different subtypes may be characterized by different behavioral symptoms [70]. Nonetheless these findings provide valuable insights into the predictors associated with QoL of NH residents with moderate to severe dementia. A notable strength of this study was the inclusion of a range of predictors, as well as the use of the Qualidem, which facilitated differentiation between a range of domains of QoL [71].

In conclusion, depression, agitation, and apathy are the most important predictors associated with a lower QoL. Additionally, there is some evidence that male gender and certain comorbid conditions (most prominently neurological diseases) were associated with aspects of QoL, highlighting the multidimensionality of QoL and the importance of assessing multiple predictors. These results suggest that special attention should be given to persons with mood and behavioral problems and comorbid conditions, as these persons may be at the greatest risk of reduced QoL. The results are clinically relevant and offer an important starting point for treatment, because unlike other factors, such as cognition and demographic factors, mood and behavior can be influenced with non-pharmacological interventions. Future research should examine the effectiveness of therapeutic interventions targeting mood and behavioral problems combined with timely treatment of comorbid conditions as possible means to improve the QoL of NH residents with dementia. The domains of QoL, with their own specific relationships, should be taken into account when selecting non-pharmacological and person centered interventions to improve QoL in persons with dementia. There is need for further research to assess the influence of comorbid conditions and gender on QoL, and how these predict future QoL.

## Supporting information

S1 File Dataset paper QoL. (SAV)
